# Antibacterial activity of multi-metallic (Ag–Cu–Li) nanorods with different metallic combination ratios against *Staphylococcus aureus*

**DOI:** 10.1186/s13104-023-06284-4

**Published:** 2023-02-28

**Authors:** Rabeah Y. Rawashdeh, Ghassan Qabaja, Borhan Aldeen Albiss

**Affiliations:** 1 Department of Biological Sciences, Faculty of Science, Yarmouk University, Irbid, Jordan; 2 Center for Research Excellence in Nanobiosciences, University of North Carolina at Greensboro, Greensboro, NC 27412 USA; 3 Institute of Nanotechnology, Jordan University of Science and Technology, Irbid, 22110 Jordan

**Keywords:** AgCu NRs, AgCuLi NRs, Bimetallic NRs, Trimetallic NRs, Antibacterial activity

## Abstract

**Objective:**

Because of the need to extensively study the synergistic activity of metallic nanoparticles, this study aimed to evaluate the antibacterial activity of mixed metallic nanoparticles, made by differing the weight mixing ratio. We prepared multi-metallic nanorods (NRs) by chemical reduction method, with different ratio combinations of silver Ag and copper Cu, two main batches of nanorods were produced: bimetallic mix made only of Ag–Cu, and trimetallic mix made of Ag–Cu and lithium Li, AgCu NRs and AgCuLi NRs respectively. NaOH was used in the synthesis for the co-reduction of salt precursors. Ag percentage was varied from 10 to 90% in bimetallic NRs but in the trimetallic NRs, which has a fixed ratio of Li (10%), the percentage of silver precursor was from 10 to 80%. The presence of metals was confirmed by energy dispersive X-rays (EDX) analysis. Ion release was detected using inductively coupled plasma spectrometer (ICP) and the values showed that NRs are effective source for ion supply for up to 24 h. The antibacterial activity of metallic NRs was tested against *Staphylococcus aureus* using Bauer Kirby method.

**Results:**

The bi-synergistic mix of Ag and Cu generates more ions than the tri-synergistic mix of Ag, Cu, and Li. Nevertheless, the later was more efficient and showed higher antibacterial activity at lower concentrations. This effect is less likely to be attributed to modality of ion release. Indeed, the results of our work suggest that besides ion release, alloyed nanorods themselves are toxic and the trimetallic mix exhibited more biocidal activity, specifically at Ag salt concentrations of 30%, 50% and 70%.

**Supplementary Information:**

The online version contains supplementary material available at 10.1186/s13104-023-06284-4.

## Introduction

Silver nanorods have medical usage as antimicrobial agents for wound and injuries treatment as well as against drug resistance infection [1–3]. Drug delivery and anticancer therapy are other developing applications of silver nanorods [4–6] their extensive use in therapeutic approaches is due to their genuine physiochemical properties [7]. Similarly, copper nanorods have received considerable attention for use in antimicrobial applications [8, 9]. Hydrothermal, sol gel synthesis, chemical reduction and other bottom-up synthesis approaches generate nanoscale metal materials that differ in size and morphology, however scientists routinely use the term nanorods to include all the other forms and shapes, such as nanospheres and nanorods.

Antibacterial reactivity of nanorods depends on shape and size along other properties [10, 11]. A wide antibacterial spectrum of silver nanorods was reported in literature, where nanorods inhibited the growth of both gram-positive and gram-negative bacteria [1, 6]. A comparative study showed that silver nanorods are more reactive biocides against *S. aureus* and other tested bacteria than copper nanorods [12]. The antibacterial behavior of metallic nanorods is complex and is stemmed from the combined effect of ions and nanorods [13]. It was demonstrated that biocidal activity of metallic nanorods results from the interaction of nanorods themselves with cellular and subcellular structures [11]. Silver nanorods exposed bacterial cells revealed breakage in DNA and higher levels of oxidative molecules [7]. Additional research needs to be done to ascertain what type of entity is more influential in antibacterial activity; nanorods or their corresponding ions. One of the important antibacterial assays, that is widely used, is the disk diffusion assay, known as the Kirby-Bauer test [1, 9].

While there is a growing knowledge of the antibacterial activity of monometallic nanorods, little information is available of synergistic antibacterial effect of multi element nanorods. CuPd, AuPd, FePd, and AgCu are examples of multi metallic nanorods having unique, magnetic, optical, physicochemical and antibacterial properties, compared with pure monometallic nanorods [14–17]. Lithium is not commonly used in the fabrication of nanorods although its antibacterial properties have been reported recently [18].

Bimetallic alloys made of silver copper, AgCu nanoparticles, AgCu NPs, are commonly synthesized by chemical method using different reducing agents and salt precursors [19]. Additionally, varying elemental concentration of silver copper, AgCu, NPs is another parameter to be used to enhance their antibacterial effectiveness. AgCu nanorods made of equal concentration ratios of metal precursors exhibited complete inhibition of *Escherichia coli* (*E. coli)* growth [15].

In this work chemically produced rod-shaped metallic nanorods were made with two and three metal combinations and at different initial concentration ratios. The relative concentration ratios of precursors of Ag and Cu was changed to prepare different suspensions of (AgCu NRs). Likewise, Ag, Cu salt ratio percentage was also changed for the preparation trimetallic NRs (AgCuLi NRs), but Li salt percentage was fixed. The aim of this study is to screen the different synergetic nanorods to find out the efficient ratio combination of AgCu to be used as antimicrobial agents.

## Methods

### Chemical reduction synthesis of silver and copper nanorods

All the chemicals used in this work were analytical grade. Copper sulfate pentahydrate (CuSO_4_.5H_2_O), Silver nitrate (AgNO_3_), Lithium sulfate (Li_2_SO_4_) were the metal salt precursors used to produce metallic nanorods, NRs. Only the first two salts were used to produce bimetallic NRs. Colloidal suspensions of heterogeneous metallic nanorods were produced in a typical single step reduction according to a method of Wang et al., 2019 [20], with slight modification. The weighed amounts of CuSO_4_.5H_2_O and AgNO_3_ were dissolved in 50 ml d. H_2_O, in a flask, and stirred with a magnetic stirrer for 10 min. For the preparation of trimetallic mixture a concentration of 0.03 mol/l of Li_2_SO_4_ was used for the synthesis of all lithium-based NRs. Each metal was represented by weight percentage. Calculation was done as follows: the weight of metal in the sample divided by the weight of all metals in the sample multiplied by 100.

For preparing NRs made of silver copper and lithium (AgCuLi NRs), concentration of Li precursor is summed up to the total concentration of metal precursors. 10% and 80% were the lowest and highest silver precursor percentage in Lithium based NRs (AgCuLi NRs). While for lithium free NRs (AgCu NRs), 10% and 90% were the lowest and the highest silver percentage respectively. (Different nanorods samples are designated NP1, NP2, NP3…, NP8).

After dissolving metal salts, the solution was transferred to a spherical flask mantle followed by one time addition of a freshly prepared solution of sodium hydroxide (NaOH) at a 4 M concentration. This solution has a dual role: for the dissociation of silver sulphate solid at the beginning of synthesis reaction as well as to act as a reducing agent for nanorods synthesis. The solution was stirred rapidly using mechanical stirrer for 10 min. The mixture was then heated, using heating mantle, to 100 °C for 1 h with permanent stirring. (Additional file 1: Fig. S1). Metal ions in solution reduced simultaneously into metal atoms, which mediate nucleation and nanorods growth. These two stages lead to the formation of metal nanorods. Formation of nanorods is indicated by a color change of mixture into dark brown. After finishing the reduction reaction and then cooling to room temperature, the metal precipitate was collected and washed with distilled water (5 times). Probe sonication was done for one hr. Finally, the collected pellet was dried at room temperature to get nano powder (the powder was stored in dark at ambient condition). Stock suspension was prepared by dispersing nano powder in d. H_2_O, then vertexing for 5 min at room temperature, followed by sonication for up to 5 min. Stock suspension was diluted using d. H_2_O into the following concentrations: 1, 5, 10, 20 µg/ml and higher concentrations at 1 and 5 mg/ml were prepared as well. NRs stock and diluted suspensions were freshly prepared in 50 ml conical centrifuge tubes. All tubes were wrapped with aluminum foil, to prevent light effect.

### Characterization of metallic NRs using scanning electron microscope/energy dispersive X ray, SEM/EDX

Metallic nanorods were characterized using scanning electron microscope (SEM) with energy dispersive X rays (EDX). The images were obtained by SEM (Hitachi S-4800, Hitachi Ltd Tokyo Japan). For SEM imaging, nonpowered was further dried in oven at 50 °C, then samples were mounted on stubs using conductive double sided carbon tape. SEM analysis was done at 15 kV acceleration voltage and approximately at 8 mm working distance. The energy dispersive x rays (EDX) technique was used for the identification and quantification of the elemental percentage in nano powder, as well as to compare the relative percent of metals in different NRs samples.

### Antibacterial activity of metallic nanorods against the growth of *Staphylococcus aureus*

The following concentrations of NRs were prepared to be tested against bacteria; 1, 5, 10, 20, 50, 100 µg/ml and 1 and 5 mg/ml. Kirby- Bauer method (disk diffusion method) was used to test the susceptibility of *Staphylococcus aureus* to the produced NRs. Filter papers (Whatman No.1 filter paper) were cut into disks and sterilized by autoclaving. Müller–Hinton agar media (HiMedia Laboratories), were inoculated with bacterial broth (*S. aureus*). Prepared disks were immersed with shaking in NRs suspension for 1–2 min at room temperature, then they were pressed gently down onto the agar. Plates were allowed to dry for 5–15 min at room temperature. Disks impregnated with gentamicin was used as the positive control and applied at various concentrations at 10, 50 and 100 µg/m, and at higher concentrations as well (1 and 5 mg/ml). Plates were incubated at 37 °C for 24 h. The diameter (mm) of the zone of inhibition was measured in millimeter and recorded for each disk.

### Measurements of silver and copper released from nanorods using ICP

**Fig. 1:**
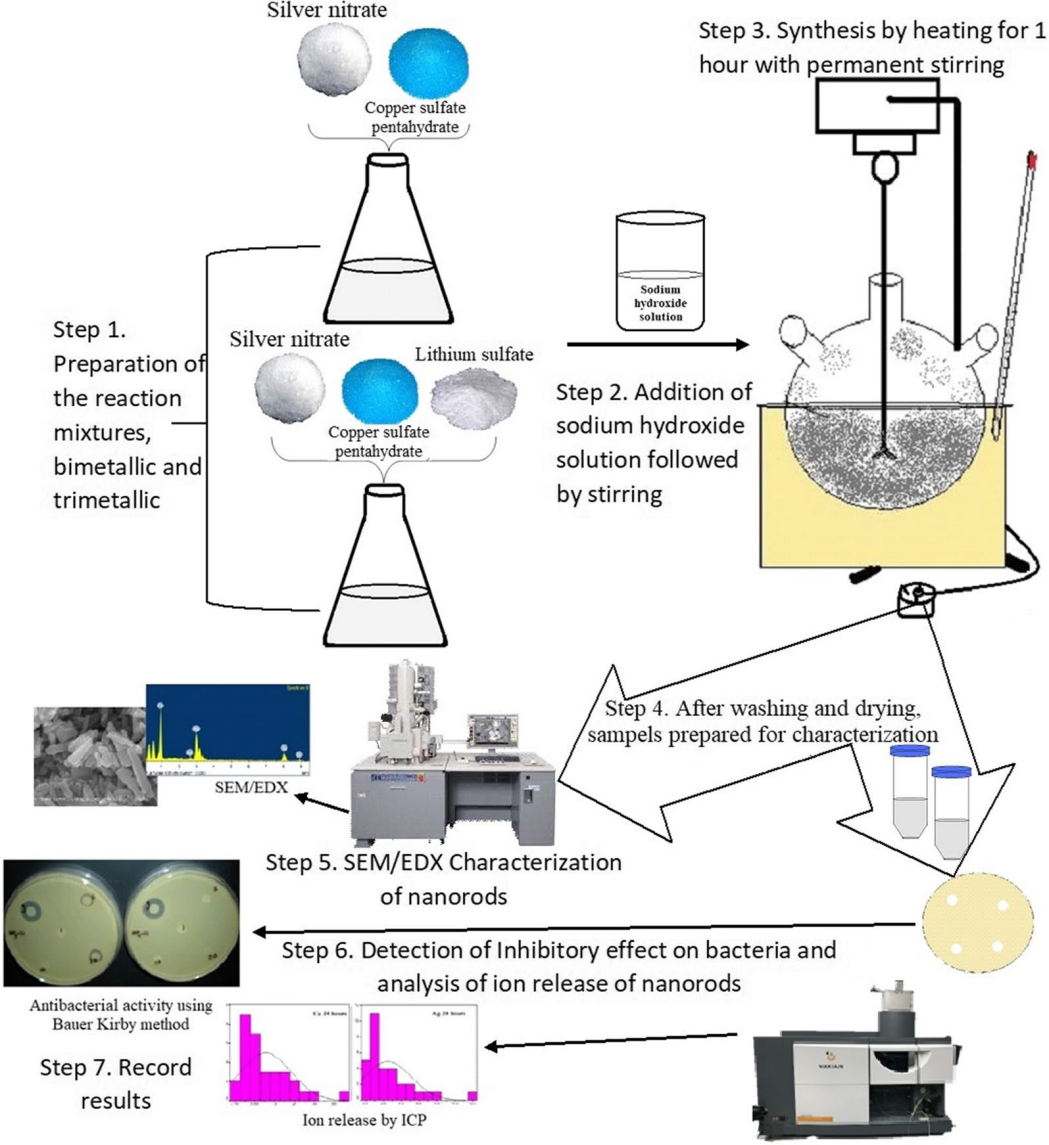
Schematic diagram of the synthesis, characterization and biological activity of multi-metallic nanorods

Ion release was measured using ICP-OES (Inductively Coupled plasma- Optical Emission Spectrometer, Varian 710-ES). For that purpose, nano metallic suspensions were freshly prepared with different concentrations; 1, 5, 10 and 20 µg/m. The first reading was taken after 2 h, NRs suspensions were kept at ambient conditions and measured again after 24 h. Before each ICP reading, centrifugation was done at 5600 ×*g* for 20 min. After centrifugation, nanorods were concentrated at the bottom of the tube. Supernatant solution was carefully removed using a syringe for ICP analysis. Working calibration standards for each element were prepared and used every time prior to sample measurement. A schematic diagram of the study methodology is shown in Fig. 1

### Statistical analysis of ions release

Statistical test (SPSS software version 11.5) was used to test if there are differences in ion release between two groups of nanorods having the same ratio of silver to copper; lithium-based NRs and lithium free NRs. Skewness values for indicate the non-symmetry for the distributions of all the released ions.

The null hypothesis of the Mann Whitney test is that the medians of the two groups are the same,

H_0_; median _with Li_ = median _without Li_.

H_1_: median _with Li_ ≠ median _without Li_.

If P > 0.05 hypothesis is accepted, if P < 0.05 hypothesis is rejected, and it can be concluded that the two groups are significantly different. ICP-OES data were reported using graphs to facilitate observation of different results.

## Results

### Characterization of metallic NRs using SEM/EDX

**Fig. 2:**
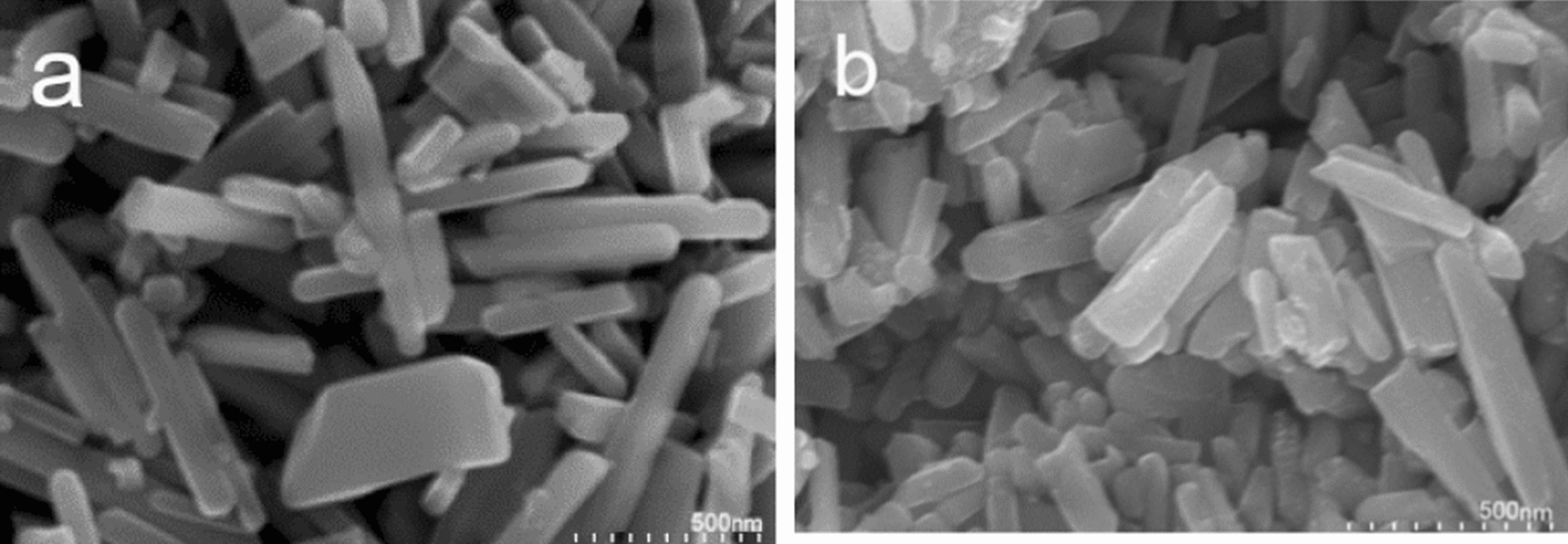
SEM images of the produced metallic nanorods: **a** Ag–Cu NRs, **b** Ag–Cu–Li NRs

**Fig. 3:**
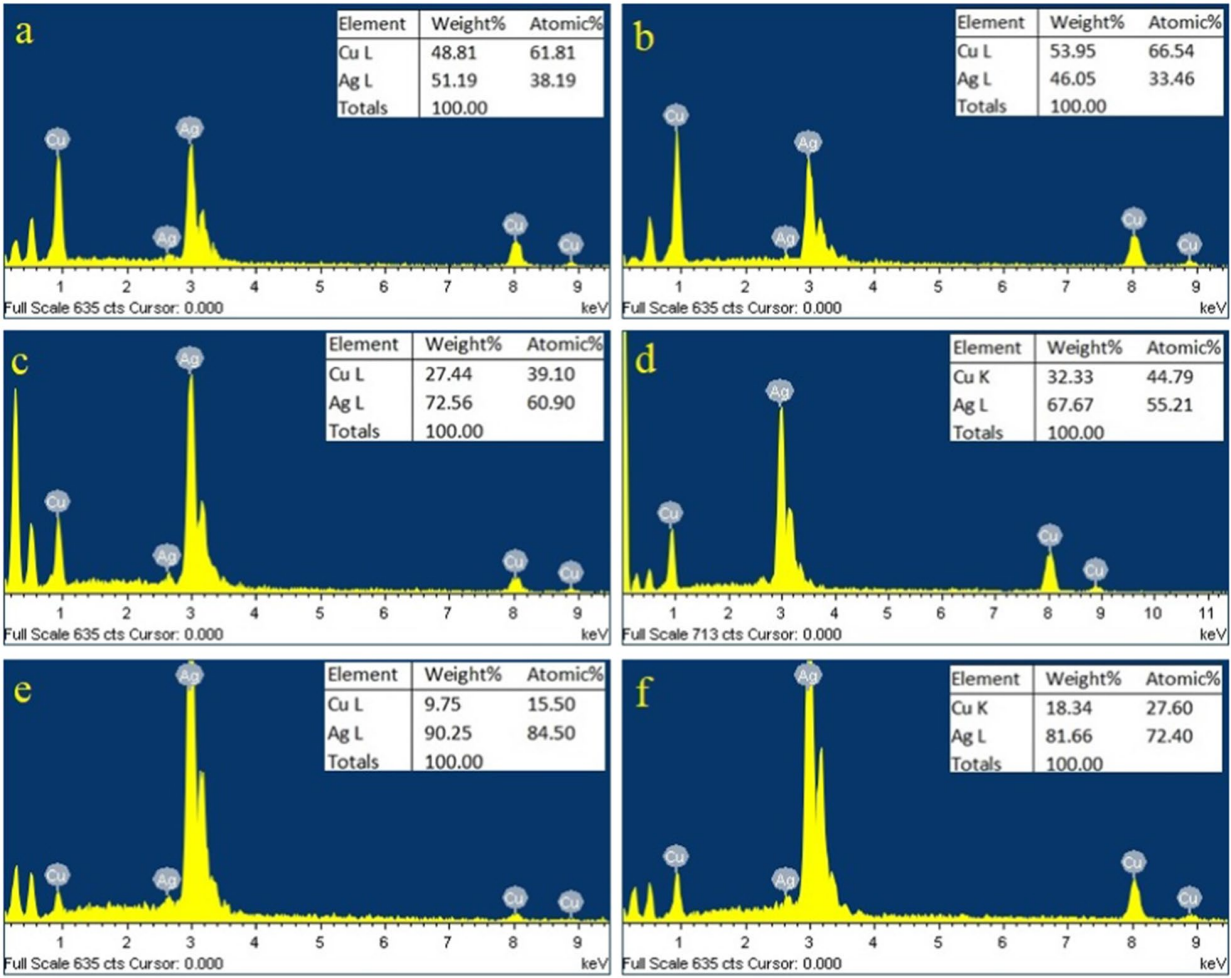
EDX spectrum of synthesized AgCuLi NRs (**a**, **c**, **e**) and AgCu NRs (**b**, **d**, **f**) with the different combinations of silver and copper (**a**: Ag_30_ Cu_60_, **b**: Ag_30_ Cu_70_, **c**: Ag_50_Cu_50_, **d**: Ag_50_Cu_40_, **e**: Ag_70_Cu_30_, **f**: Ag_70_Cu_20_)

Images of the scanning electron microscope of the synthesized bimetallic NRs and trimetallic NRs show rod shaped morphology (Fig. 2). Nanorod’s diameter was less than 100 nm and their lengths range from 200 to 800 nm. AgCu NRs and AgCuLi NRs show same morphology with no differential features in size or shape. Energy dispersive x rays (EDX) technique was used to determine the relative elemental composition in nanorods by measuring the intensity of the characteristic emitted X rays. The components of the first batch of NRs are two metal colloids (Ag and Cu) and the other batch is made of three metal colloids (Ag, Cu and Li). The EDX analysis of bimetallic and trimetallic nanorods shows an intense silver signal at 3 keV and copper signals at 1 and 8 keV (Fig. 3). All EDX spectra obtained confirmed the presence of elemental silver and copper in examined nanorod samples, also proved the purity of the obtained samples of AgCu and AgCuLi NRs. Table 1 shows all ratio combinations of mixed colloidal NRs and the elemental analysis of each metal (EDX output). The analytical data are available as supplementary material (Additional file 1: Table S1).

**Table 1:** EDX elemental composition analysis of silver and copper detected in different combination ratios of bimetallic (AgCu) and trimetallic (AgCuLi) NRs

	AgCu NRs				AgCuLi NRs			
	Ag:Cu ratio		EDX		Ag:Cu ratio		EDX	
NP	Ag	Cu	Ag	Cu	Ag	Cu	Ag	Cu
1	10	90	17.6	82.4	10	80	11.4	88.6
2	20	80	30.6	69.4	20	70	35.4	64.6
3	30	70	46.4	53.6	30	60	51.03	48.97
4	40	60	54.4	45.5	40	50	67.7	32.4
5	50	50	67.1	32.9	50	40	72.6	27.4
6	60	40	68.8	31.3	60	30	77.3	22.7
7	70	30	83.1	16.9	70	20	82.6	17.4
8	80	20	91	8.9	80	10	93.9	6.03
9	90	10	95.4	4.6				

### Antibacterial activity of silver copper nanorods against the growth of *Staphylococcus aureus*

**Fig. 4:**
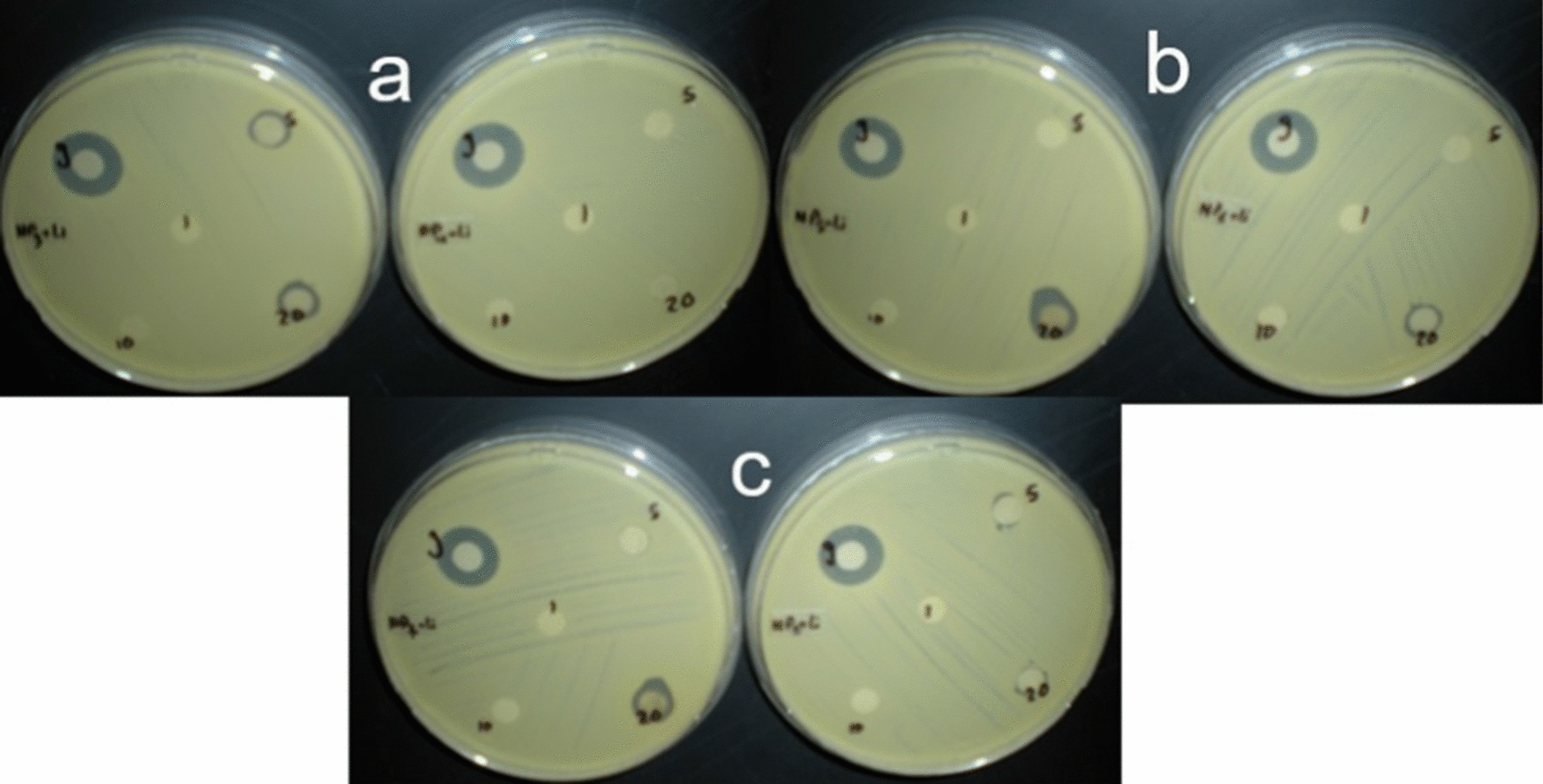
Results of Bauer Kirby method for AgCuLi NRs. The following concentrations of NRs were tested in one plate; 1, 5, 10, 20 µg/ml. Gentamicin is represented by g. **a** Represents NP3 (Ag_30_: Cu_60_) at left and NP4 (Ag_40_: Cu_50_) at right. **b** Represents NP5 (Ag_50_: Cu_40_) at left and NP6 (Ag_60_: Cu_30_) at right. **c** Represents NP7 (Ag_70_: Cu_20_) at left and NP8 (Ag_80_: Cu_10_) at right

Kirby-Bauer test showed no difference between the different AgCu NRs with different initial ratio combinations of silver and copper. At 1 mg/ml concentration trimetallic showed higher antibacterial activity than the bimetallic NRs. Trimetallic NRs exhibited antibacterial activity against bacteria for almost all ratio combinations (Additional file 1: Figs. S2, S3), zone of inhibition was more than 10 mm for higher concentration of NRs, 1 and 5 mg/ml. AgCuLi NRs at initial ratios of Ag_30_ Cu_60_, Ag_50_Cu_40_, and Ag_70_Cu_20_ showed biocidal activity at 20 µg/ml (Fig. 4).

### ICP Measurements of silver and copper release from NRs and statistical analysis

**Fig. 5:**
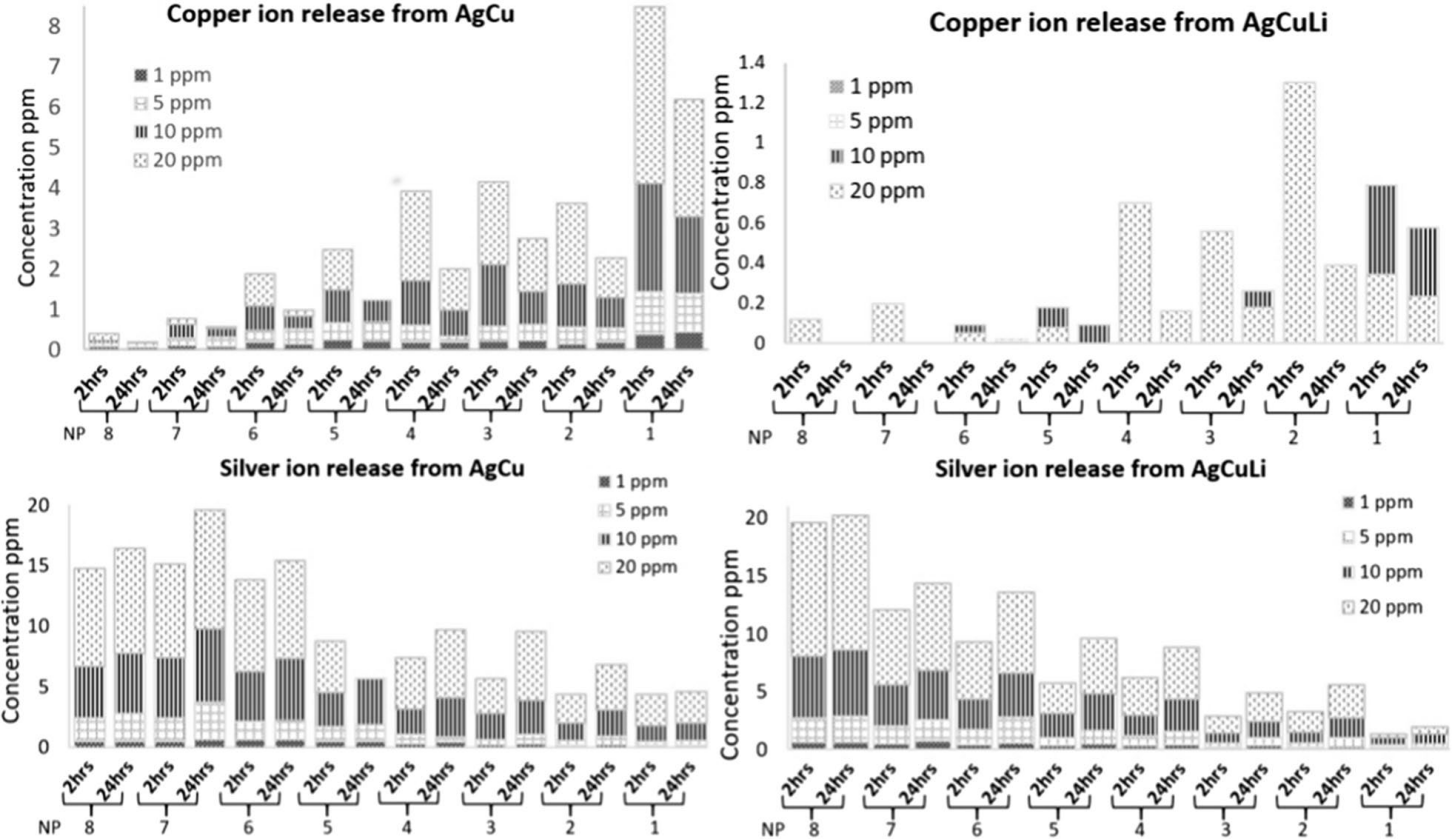
Differences in Cu ions release, and Ag ions release of lithium free nanorods (AgCu NRs) and of lithium-based nanorods (AgCuLi NRs), ion release was measured after 2 and 24 h. The top graphs for Cu ions release of AgCu NRs and AgCuLi NRs. The bottom graphs for Ag ions release of AgCu NRs and AgCuLi NRs

Descriptive analysis was done for the release values of Ag and Cu after 2 h and 24 h of preparation of NRs suspension (Additional file 1: Table S2). Results showed that more silver and copper released from bimetallic NRs (Fig. 5). To find out if the difference is significant, Mann Whitney test U test was conducted to evaluate the hypothesis that ion release from NRs with lithium is different than the release from NRs without Li (Table 2).

**Table 2:** Results of Mann Whitney U test of ion release after different times of AgCuLi NRs and AgCu NRs

Metal measured	Release time (hrs.)	Type of metallic NRs	N^a^	Sum of ranks	Mean rank	Mann–Whitney U	Asymp. Sig. (2-tailed)
Cu	2	Tri	32	796	24.8	268.00	0.00
		Bi	36	1550	43.0		
	24	Tri	32	765.5	23.9	237.50	0.00
		Bi	36	1580.5	43.9		
Ag	2	Tri	32	992.0	31.0	464.00	0.169
		Bi	36	1354.0	37.6		
	24	Tri	32	1050.0	32.8	522.00	0.50
		Bi	36	1296.0	36.0		

^a^Size of the sample

The results were significant for copper. z = − 3.791. P < 0.05 after two hrs, and z = − 4.167. P < 0.05 after 24 h. Thus, there was significant difference (P < 0.05) in Cu ions released from NRs with and without lithium, while for Ag ions there was no significant difference between NRs with and without Li (P > 0.05).

## Discussion

The variation in chemistry of nanorods synthesis is extensive and results in differences in size, morphology, and performance [18]. In this study, silver copper synergistically mixed at a range of ratios in bimetallic (AgCu) alloys and trimetallic (AgCuLi) alloys. For the metal salts solution, the predominant Ag entity is silver sulfate (Ag_2_SO_4_) which results from displacement reaction at room temperature. Copper and lithium ions (Cu^+2^ and Li^+^) are strongly soluble in water and form electrolytes.

The use of high molar concentration of sodium hydroxide was important in nanorods synthesis. One of its reacting roles is the dissociation of low soluble silver sulphate at the beginning of synthesis reaction. Also, sodium hydroxide acts as a reducing agent for nanorods synthesis. It was reported in literature that AgCu NPs with a pH of 7 to 9 have enhanced properties and activities. The use of NaOH in the synthesis increases the pH of the solution, and according to a study of Kubanalievich et al. [21] It has been established that the most highly dispersed nano metals, of silver and copper, were formed in an alkaline medium. Also, high pH of synthesis reaction yields both spherical and rod-like Ag nanorods, which form as a result of fast reduction rate of the precursor [22]. A study done revealed that the higher the pH is the more the antibacterial efficiency is [23].

SEM micrographs of the produced NRs showed rod shaped because of the use of NaOH in synthesis, this results in fast and seedless reduction reaction [24, 25]. Few studies revealed the bio-functional dependence of nanorods on shape and geometry [10, 24, 26]. A previous study showed that silver nanorods have greater surface area therefore showed higher antibacterial activity than silver nanoparticles [27]. Nanorods were assembled in large aggregates, such aggregates form because of weak Brownian motion [28]. The physiochemical properties of colloidal silver and copper NPs, particularly for systems that are made chemically, are well documented in literature. Their consisted parameters indicate their stability [29]. Previous XRD studies revealed the crystalline nature of multi-metallic nanoparticles such as the alloyed silver copper nanoparticles [15, 30]. Nanomaterial behavior depends significantly on metal choice, synthesis approach and the applied concentration. In order to overcome cellular toxicity limitation, which is one of the most prominent limitations, of the application of metal-based nanomaterial, less reactive metals were selected over other metals for the preparation of nanorods, silver and copper are less reactive than zinc. Also, a working concentration of 20 ppm was reported to be safe and biocompatible.

For structural compositional analysis, energy dispersive x rays (EDX) is a technique used in literature for detecting metal percent in metallic nanorod systems [31]. Likewise, EDX was used in this work for compositional analysis, and it showed the presence of metals in all types of nanoalloys. Detected silver and copper contents correlate to the metallic input content, in other words, increasing metal weight percentage in nano powder results in higher levels of metal content. The produced metallic NRs, made with a range of elemental ratios, were tested against *Staphylococcus aureus*. The concentration of antibacterial nanorods is a critical determining parameter in their efficacy to inhibit bacterial growth. Also, the antimicrobial activity of nanoparticles depends on a greater aspect on their size, where smaller nanoparticles have larger surface to volume ratio, thus they have greater antibacterial activity [10, 32]. Clear inhibition zone was detected around NRs disks indicating antibacterial activity, this was recorded at a concentration of 1 mg/ml for all types of metallic NRs. Trimetallic NRs showed antibacterial activity at lower concentrations.

ICP analysis revealed that lithium containing NRs are less prone to release metals. This observation was not reported in literature. The results of the current study provided that the degree of metal combination govern ion release of nano alloys. For instance, bimetallic NRs release more silver and coper relatively to trimetallic NRs. Copper ions were significantly released at higher level. However, trimetallic NRs exhibited higher antibacterial activity than the bimetallic NRs, particularly at the following silver percentages: 30, 50 and 70.

Although the antibacterial effect is associated with ion release, silver nanorods themselves exert biocidal effect at more than one bio-target [33–35]. The previous results can also be attributed to the fact there are many anti-bacterial mechanisms exerted by nanometals, and it is not necessarily associated with higher number of released metallic ions [12, 35]. Trimetallic alloys nanorods (Silver copper and lithium) were effectively more toxic. This due to incorporating lithium, the reactive toxic element, in the nano metallic mix. Lithium nanorods rendered more toxicity on bacteria. The compromised dual antibacterial mechanisms, via metal ions and the dynamic toxic nanorods, mediate toxicity of nano metallic nanorods.

## Conclusions

Bimetallic (AgCu) and trimetallic (AgCuLi) nanoalloys were synthesized using one step co-reduction method. Bacteria used in this study were more susceptible to lithium containing nanoalloys at a concentration as low as 20 µg/ml, at the following metallic ratios of silver copper; (Ag_30_:Cu_60_), (Ag_50_:Cu_40_), and (Ag_70_: Cu_20_). Multi-metallic composition of the prepared nanorods denotes heterogeneity in their antibacterial mechanism. Also, results revealed that the more powerful antibacterial lithium-containing nanorods were less prone to release metals, this indicates that the basis of antibacterial effectiveness depends not only on ion release but also on the synergistic effect of the interacting mixed nanorods. Our results suggest that the toxic impact of nanoparticulate systems depends on structural heterogeneity which stem from the difference and arrangement of the elemental constituents.

## Limitations

Many techniques could be used to provide full characterization of the chemical and physical properties of the synthesized nanorods. Zeta potential and X ray diffraction are techniques used respectively to measure surface charge and the crystal structure of nanorods.

## Supplementary Information

**Additional file 1: Fig. S1.** A sketch of the apparatus set that was used for the chemical synthesis of nanorods. **Fig.S2.** and **Fig. S3.** Results of Bauer Kirby method. **Table S1.** The measurement of ion release by inductively coupled plasma. **Table S2.** The descriptive analysis for ion release.

## Data Availability

Data generated or analyzed during this study are included in the supplementary file. Additional data regarding synthesis and preparation of metallic nanoparticles may be available from the corresponding author upon request.

## Abbreviations

AgCu NRs: AgCu nanorods
AgCuLi NRs: AgCuLi nanorods
Bimetallic NRs: Bimetallic nanorods
Trimetallic NRs: Trimetallic nanorods
NPs: Nanoparticles
*S. aureus*: *Staphylococcus aureus*
SEM: Scanning electron microscope
EDX: Energy dispersive X-rays
ICP: Inductively coupled plasma spectrometer
*E. coli*: *Escherichia coli*
Hrs: Hours
Min: Minutes
