# Supplementary material for: Antibacterial activity of multi-metallic (Ag–Cu–Li) nanorods with different metallic combination ratios against Staphylococcus aureus
Source: BMC Res Notes. 2023 Feb 28;16:23. doi: 10.1186/s13104-023-06284-4 (PMC9976529; doi:10.1186/s13104-023-06284-4)
Supplement: Supplementary file 1 — Additional file 1: Fig. S1. A sketch of the apparatus set that was used for the chemical synthesis of nanorods. Fig.S2. and Fig. S3. Results of Bauer Kirby method. Table S1. The measurement of ion release by inductively coupled plasma. Table S2. The descriptive analysis for ion release. [file 13104_2023_6284_MOESM1_ESM.docx]

**Additional file**

- -
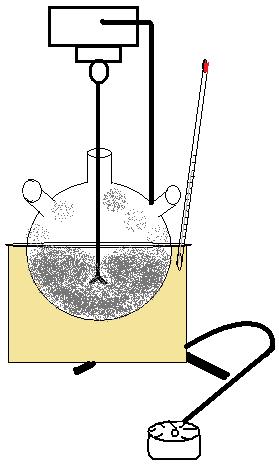


Mechanical stirrer

Thermometer

Mixture

Sand

Heat monitor

Fig. S1: the set used for mixing and heating the components of NPs


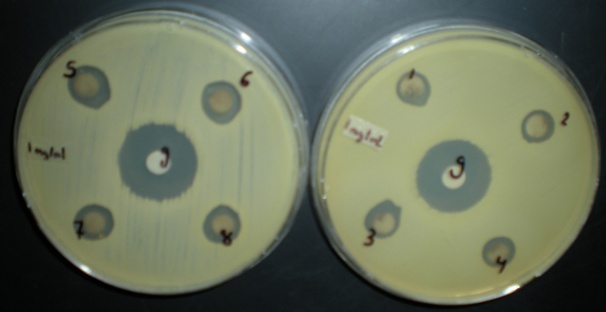


Fig. S2: Results of Bauer Kirby method for higher concentrations of AgCuLi NPs: 1mg/ml. NP1, NP2, NP3 and NP4 at the right plate. NP5, NP6, NP7 and NP8 in the left plate.

A

B


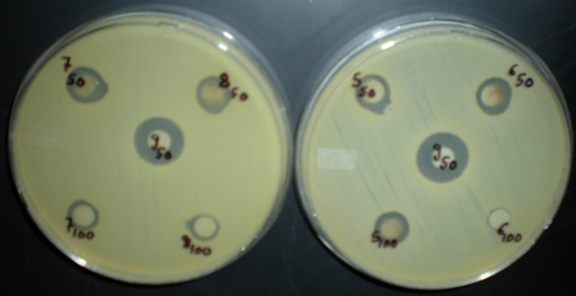


Fig. S3: Results of Bauer Kirby method of AgCuLi NPs using 50µg/ml at the top of each plate and 100µg/ml at the bottom of each plate. NP5, NP6 at the right plate and NP7, NP8 at the left plate. Gentamicin is represented by g.

A

B

Table S1: ICP measurements of Ag and Cu release at different time intervals; 2 & 24 hours using different dilutions of NPs; 1, 5, 10 & 20 µg/ml

| **Dilution**  **µg/ml** | **NPs with Li** | | | | | **NPs without Li** | | | | |
| --- | --- | --- | --- | --- | --- | --- | --- | --- | --- | --- |
|  | **Input in%** | **ICP (2hrs)** | | **ICP (24hrs)** | | **Input in%** | **ICP (2hrs)** | | **ICP (24hrs)** | |
|  |  | Cu  µg/ml | Ag  µg/ml | Cu  µg/ml | Ag  µg/ml |  | Cu  µg/ml | Ag  µg/ml | Cu  µg/ml | Ag  µg/ml |
| 1 | 1  10 Ag  80 Cu | 0.004 | 0.16 | 0.005 | 0.174 | 1  10 Ag  90 Cu | 0.37 | 0.1 | 0.44 | 0.11 |
| 5 |  | 0.16 | 0.22 | 0.153 | 0.295 |  | 1.06 | 0.4 | 0.95 | 0.42 |
| 10 |  | 0.79 | 0.61 | 0.58 | 0.897 |  | 2.66 | 1.3 | 1.9 | 1.5 |
| 20 |  | 0.344 | 0.33 | 0.233 | 0.61 |  | 4.38 | 2.57 | 2.9 | 2.6 |
| 1 | 2  20 Ag  70Cu | -0.01 | 0.19 | 0.019 | 023 | 2  20 Ag  80 Cu | 0.15 | 0.14 | 0.18 | 0.18 |
| 5 |  | 0.23 | 0.35 | 0.192 | 0.76 |  | 0.41 | 0.44 | 0.36 | 0.77 |
| 10 |  | 0.76 | 0.93 | 0.30 | 1.71 |  | 1.07 | 1.44 | 0.74 | 2.1 |
| 20 |  | 1.3 | 1.77 | 0.39 | 2.9 |  | 1.99 | 2.36 | 0.99 | 3.8 |
| 1 | 3  30 Ag  60 Cu | -0.03 | 0.19 | -0.04 | 0.34 | 3  30 Ag  70 Cu | 0.20 | 0.22 | 0.23 | 0.27 |
| 5 |  | 0.07 | 0.34 | 0.08 | 0.699 |  | 0.38 | 0.44 | 0.397 | 0.76 |
| 10 |  | 0.38 | 0.89 | 0.26 | 1.36 |  | 1.52 | 2.1 | 0.81 | 2.84 |
| 20 |  | 0.56 | 1.5 | 0.18 | 2.5 |  | 2.03 | 2.91 | 1.3 | 5.7 |
| 1 | 4  40 Ag  50 Cu | 0.0005 | 0.29 | 0.0007 | 0.39 | 4  40 Ag  60 Cu | 0.18 | 0.28 | 0.18 | 0.42 |
| 5 |  | 0.14 | 0.86 | 0.0999 | 1.15 |  | 0.41 | 0.79 | 0.145 | 0.46 |
| 10 |  | 0.42 | 1.79 | 0.15 | 2.8 |  | 1.12 | 2.1 | 0.66 | 3.2 |
| 20 |  | 0.7 | 3.31 | 0.16 | 4.51 |  | 2.2 | 4.2 | 1.01 | 5.6 |
| 1 | 5  50 Ag  40 Cu | -0.03 | 0.31 | -0.04 | 0.5 | 5  50 Ag  50 Cu | 0.25 | 0.51 | 0.21 | 0.46 |
| 5 |  | 0.022 | 0.68 | 0.054 | 1.11 |  | 0.41 | 1.2 | 0.47 | 1.4 |
| 10 |  | 0.18 | 2.1 | 0.091 | 3.24 |  | 0.82 | 2.8 | 0.55 | 3.83 |
| 20 |  | 0.08 | 2.69 | -0.062 | 4.8 |  | 1.0 | 4.3 | Nd | Nd |
| 1 | 6  60 Ag  30 Cu | -0.02 | 0.41 | -0.03 | 0.54 | 6  60 Ag  40 Cu | 0.18 | 0.62 | 0.15 | 0.61 |
| 5 |  | 0.04 | 1.34 | 0.023 | 2.26 |  | 0.30 | 1.5 | 0.36 | 1.6 |
| 10 |  | 0.09 | 2.6 | 0.001 | 3.82 |  | 0.59 | 4.1 | 0.31 | 5.1 |
| 20 |  | 0.05 | 5.0 | -0.11 | 6.95 |  | 0.81 | 7.6 | .15 | 8.12 |
| 1 | 7  70 Ag  20 Cu | -0.05 | 0.51 | -0.1 | 0.75 | 7  70 Ag  30 Cu | 0.09 | 0.51 | 0.08 | 0.66 |
| 5 |  | -0.03 | 1.52 | -0.012 | 1.8 |  | 0.19 | 1.93 | 0.23 | 3.1 |
| 10 |  | 0.01 | 3.6 | -0.06 | 4.3 |  | 0.35 | 4.96 | 0.20 | 6.02 |
| 20 |  | 0.197 | 6.5 | -0.08 | 7.5 |  | 0.14 | 7.7 | 0.05 | 9.8 |
| 1 | 8  80 Ag  10 Cu | -0.05 | 0.63 | -0.062 | 0.62 | 8  80 Ag  20 Cu | 0.079 | 0.499 | 0.05 | 0.52 |
| 5 |  | -0.07 | 2.08 | -0.052 | 2.3 |  | 0.088 | 1.96 | 0.12 | 2.3 |
| 10 |  | 0.01 | 5.35 | -0.11 | 5.7 |  | 0.05 | 4.2 | -0.005 | 4.91 |
| 20 |  | 0.12 | 11.6 | -0.1 | 11.6 |  | 0.17 | 8.13 | 0.01 | 8.67 |
| ______________________________________________________________________________________________________________________________________ | | | | | | 9  90 Ag  10 Cu | 0.039 | 0.94 | 0.03 | 1.4 |
|  |  |  |  |  |  |  | 0.05 | 2.4 | 0.05 | 3.3 |
|  |  |  |  |  |  |  | 0.03 | 5.7 | -0.02 | 6.17 |
|  |  |  |  |  |  |  | 0.21 | 11.8 | 0.06 | 13.0 |

Table S2: Descriptive analysis for the Ag and Cu ions released after 2 & 24 hours, Skewness and Kurtosis values are included to evaluate the normality and symmetry of the distributions.

| **NPs** | **element** | **Time (hours)** | **N^a^** | **Min^b^** | **Max^c^** | **Mean** | **Std^d^** | **Variance** | **Skewness** | | **Kurtosis** | |
| --- | --- | --- | --- | --- | --- | --- | --- | --- | --- | --- | --- | --- |
|  |  |  |  |  |  |  |  |  | **Statistic** | **Std. Error** | **Statistic** | **Std. Error** |
| **With Li** | **Cu** | **2** | 32 | -0.07 | 1.3 | 0.198 | 0.32 | 0.099 | 1.919 | 0.414 | 3.809 | 0.809 |
|  |  | **24** | 32 | -0.11 | 0.58 | 0.07 | 0.16 | 0.03 | 1.41 | 0.414 | 2.229 | 0.809 |
|  | **Ag** | **2** | 32 | 0.16 | 11.6 | 1.897 | 2.41 | 5.791 | 2.56 | 0.414 | 7.9456 | 0.809 |
|  |  | **24** | 32 | 0.17 | 11.6 | 2.47 | 2.61 | 6.79 | 1.816 | 0.414 | 3.781 | 0.809 |
| **No Li** | **Cu** | **2** | 36 | 0.03 | 4.38 | 0.72 | 0.93 | 0.86 | 2.28 | 0.393 | 6.06 | 0.768 |
|  |  | **24** | 36 | -0.02 | 2.9 | 0.45 | 0.6 | 0.358 | 2.53 | 0.393 | 7.74 | 0.768 |
|  | **Ag** | **2** | 36 | 0.10 | 11.8 | 2.64 | 2.72 | 7.42 | 1.62 | 0.393 | 2.67 | 0.768 |
|  |  | **24** | 36 | 0.11 | 13.0 | 3.11 | 3.13 | 9.77 | 1.38 | 0.393 | 1.74 | 0.768 |

a: number of the sample. b: minimum, c: maximum, d: standard deviation
